# The acceptability of lifestyle medicine for the treatment of mental illness: perspectives of people with and without lived experience of mental illness

**DOI:** 10.1186/s12889-024-17683-y

**Published:** 2024-01-13

**Authors:** Karyn Richardson, Rachel Petukhova, Sam Hughes, Joseph Pitt, Murat Yücel, Rebecca Segrave

**Affiliations:** 1 BrainPark, Turner Institute for Brain and Mental Health, School of Psychological Sciences & Monash Biomedical Imaging Facility, Monash University, Clayton, VIC Australia; 2 QIMR Berghofer Medical Research Institute, Herston, QLD Australia; 3 Department of Psychiatry, School of Clinical Sciences, Monash University, Clayton, VIC Australia

**Keywords:** Lifestyle medicine, Behavioural interventions, Psychiatry, Acceptability, Implementation

## Abstract

**Objective:**

While lifestyle medicine can be highly effective for treating a range of mental illnesses these approaches are grossly underutilised and have not been systematically implemented into health care systems. Understanding the acceptability of lifestyle medicine is a critical first step to remediate this. This study evaluated the acceptability of lifestyle medicine relative to pharmacotherapy and psychotherapy, and explore perspectives of people with and without lived experience of mental illness.

**Methods:**

Six hundred and forty-nine adult Australian residents (62.6% female; 53.6% with a lifetime diagnosis of mental illness) completed an online survey based on the Theoretical Framework of Acceptability assessing the acceptability of lifestyle medicine, pharmacotherapy and psychotherapy for treating mental illness.

**Results:**

Most participants felt positive about lifestyle medicine (76.9%) and felt that such approaches aligned with their personal values (74.9%). They understood how lifestyle medicine worked (86.4%) and believed it would be effective (69.6%). Lived experience of mental illness was associated with greater perceived burden and lower self-efficacy to engage in lifestyle medicine activities (both *p* < 0.001). While there was a clear preference for psychotherapy and lifestyle medicine over pharmacotherapy, pharmacotherapy was perceived as least effortful (*p* < .001) and participants were least confident in their ability to engage in lifestyle medicine (*p* < 0.05).

**Conclusion:**

The findings indicate strong acceptability of lifestyle medicine for mental illness, a preference for non-pharmacological treatment approaches, and an understanding of the challenges associated with making long-term healthy lifestyle modifications amongst people who have lived experience of mental illness.

**Supplementary Information:**

The online version contains supplementary material available at 10.1186/s12889-024-17683-y.

## Introduction

Over one billion people worldwide are living with mental illness [1] and this number is continuing to rise [2]. While there has been an increase in mental healthcare expenditure [3–5] and access to traditional first-line treatments (i.e., psychotherapy and pharmacotherapy), in countries such as Australia and the US, this has not reduced the burden of mental illness or substantially improved therapeutic outcomes [6]. Mental illness continues to be among the leading causes of disability globally and is estimated to cost the global economy over $6 trillion US by 2030 [1]. A rapidly growing body of evidence indicates that lifestyle medicine approaches can be highly effective for numerous mental health indications [7–10] and are an underutilised treatment option that has the potential to offer a flexible, and empowering approach to improving mental health outcomes.

Lifestyle medicine, also known as behavioural medicine, is a branch of medicine that applies environmental, behavioural, medical, and motivational principles to promote the management of healthy lifestyle behaviours [11]. Physical exercise, sleep, diet, mindfulness meditation, and strengthening positive relationships are examples of lifestyle medicine activities with demonstrated efficacy to prevent and treat a wide variety of mental illnesses; albeit to varying degrees [7, 9, 12, 13]. For example, physical exercise and mindfulness-based interventions have been shown to be as effective as pharmacotherapy in the treatment of Major Depressive Disorder [14–17] and Generalised Anxiety Disorder [18], respectively. Clinical trials have shown that interventions targeting sleep can reduce depression severity [19–21], while the field of nutritional psychiatry [22] has provided early evidence that interventions targeting diet quality can also reduce depressive symptoms [23]. Collectively, the weight of high-quality clinical evidence has led to lifestyle modification being recommended as a first-line treatment for mental illness in international treatment guidelines by organisations such as the National Institute for Health and Care Excellence (i.e. NICE Depression in adults: treatment and management [24]), the Royal Australian and New Zealand College of Psychiatrists (i.e. RANZCP clinical practice guidelines for mood disorders [7]), and the World Federation of Societies for Biological Psychiatry (WFSBP) and Australasian Society of Lifestyle Medicine (ASLM; i.e., Clinical guidelines for the use of lifestyle-based mental health care in major depressive disorder [13]).

The value of lifestyle medicine comes not only from its capacity to treat mental illness but from its positive concurrent impacts on physical health [25]. This is particularly relevant for people living with mental illness who are twice as likely to be diagnosed with a cardiometabolic condition such as diabetes, obesity and cardiovascular disease [25–29], while those with a ‘severe mental illness’ (e.g., schizophrenia, bipolar disorder, obsessive compulsive disorder) have a 10–20 year reduction in life expectancy [30–32], largely due to these physical comorbidities. It is well established that lifestyle modification (e.g., diet, physical exercise, sleep) plays an important role in both preventing and treating cardiometabolic conditions and increasing life expectancy [25]. That these physical health benefits are unique to lifestyle medicine, as compared to psychotherapy and psychopharmacology, lends further weight to the utility of the widespread implementation of this approach.

Despite the weight of positive evidence, lifestyle-based interventions are rarely prescribed and have not been widely integrated into the mental healthcare system [9, 10]. Barriers to the wide-spread implementation of lifestyle medicine for mental illness are complex and include, but are not limited to: a lack of education and training in the prescription of effective lifestyle medicine approaches amongst practitioners [33, 34], limited funding pathways for allied health professionals to treat mental illness, and a scarcity of programs designed to support healthy lifestyle change [9]. One key ingredient to encouraging individual-level uptake and system-level implementation of lifestyle medicine is understanding how acceptable this approach is to end-users. Acceptability to end-users is important for all health treatments [35, 36], and especially for behavioural approaches which require significant sustained motivation and effort to be effective. Initiating and maintaining the level of lifestyle behaviour required to improve mental health outcomes is difficult and often more so for people living with mental illness [25]. People living with mental illness experience unique barriers to health behaviour change such as low mood, amotivation, reduced social support [37], higher rates of sleep disturbance, poor diet quality, and sedentary behaviour [25]. These distinct barriers make it particularly important to differentiate the perspectives of people with (i.e. patients) and without (i.e. potential future patients) mental illness. Evaluating the acceptability of lifestyle medicine and identifying any reservations people may have will provide insights into the value of continuing efforts to integrate lifestyle medicine alongside pharmacological and psychological approaches.

The Theoretical Framework of Acceptability (TFA [36]) is a gold-standard, empirically derived framework that outlines seven unique component constructs of acceptability: affective attitude, ethicality, burden, intervention coherence, perceived effectiveness, opportunity cost and self-efficacy (see Table 1 for definitions). It was developed via evidence synthesis to bring clarity to the concept of acceptability and enable a nuanced examination of its many facets. In the context of the current study, the TFA provided a rigorous framework upon which to investigate: 1) the acceptability of lifestyle medicine for the treatment of mental illness, 2) whether lived experience of mental illness influences the acceptability of lifestyle medicine, and 3) compare the acceptability of lifestyle medicine to psychopharmacotherapy and psychotherapy.

**Table 1:** TFA component constructs, adapted from Sekhon et al., 2017 [36]

TFA Component Construct	Definition
Affective Attitude	How an individual feels about the intervention
Burden	The perceived amount of effort that is required to participate in the intervention
Ethicality	The extent to which the intervention has good fit with an individual’s value system
Intervention Coherence	The extent to which the participant understands the intervention and how it works
Opportunity Cost	The extent to which benefits, profits, or values must be given up to engage in the intervention
Perceived Effectiveness	The extent to which the intervention is perceived as likely to achieve its purpose
Self-Efficacy	The participant’s confidence that they can perform the behaviour(s) required to participate in the intervention

## Methods

### Design

This study employed a cross-sectional survey-based research design.

### Participants

Participants included 899 Australian adults (aged 18 years and above) recruited via advertisements on social media platforms (Facebook and Twitter), online forums (Reddit) and community organisation email lists (i.e., mental health support groups). Inclusion criteria were being aged 18 years or older and residing in Australia, and no exclusion criteria were applied. Upon completion of the study survey, participants were invited to enter a prize draw to win one of three $50 grocery/department store gift vouchers to show appreciation for their effort. The current analyses excluded participants who did not complete all survey items (*n* = 250). Thus, the resulting study sample included 649 participants (62.6% female, *mean age* = 34.8 years, *SD* = 12.7). This study was conducted in accordance with the Declaration of Helsinki, approved by the Monash University Human Research Ethics Committee, and all participants provided written informed consent.

### Measures

#### Acceptability survey construction

A survey assessing the acceptability of lifestyle medicine, psychotherapy and pharmacotherapy to treat mental illness across each of the seven constructs of the TFA was developed by the research team. Survey construction and piloting was conducted using previously published methods [38, 39] and comprised the following five steps:

1. A literature review was conducted to identify words and phrasing commonly used to describe and assess each of the seven TFA constructs (e.g. Affective Attitude: like, enjoy, feel positive; Intervention Coherence: comprehensible, understand, easy to follow [38–41].
2. The research team discussed these options and achieved consensus on the most grammatically correct and readable phrasing to accurately reflect each TFA construct (see Table 2).
3. Two researchers drafted items for each TFA acceptability domain based on the agreed phrasing.
4. Items were reviewed and refined by the broader research team and consensus achieved on the wording that most accurately reflected the core conceptual meaning of the TFA constructs.
5. The survey items were piloted with five non-academic community members who provided feedback on clarity and ease of completion.

**Table 2:** Example survey items

TFA Component Construct	Key words	Example Item
Affective Attitude	“feel positive”	I feel positive about the use of lifestyle medicine activities to treat mental illness
Burden	“effort”	I think engaging in lifestyle medicine activities to treat mental illness would require too much effort
Ethicality	“my personal values”	Using lifestyle medicine activities to treat mental illness fits with my personal values
Intervention Coherence	“understand”	I understand how engaging in lifestyle medicine activities could treat mental illness
Opportunity Cost	“give up”	Engaging in lifestyle medicine activities to treat mental illness would come at a cost, it would mean giving up other things that are important to me
Perceived Effectiveness	“effective”	Engaging in regular lifestyle medicine activities would be an effective treatment for mental illness
Self-Efficacy	“confident”	If I had a mental illness, I am confident I could regularly engage in lifestyle medicine activities to treat it

The same key words and item structure was used for TFA items assessing the acceptability of pharmacotherapy and psychotherapy

Shortly after data collection Sekhon and colleagues published a validated TFA-based questionnaire designed to assess the acceptability of healthcare interventions [42]. The items in the current survey align closely with those in the validated scale (e.g. “I understand how engaging in lifestyle medicine activities could treat mental illness” vs “It is clear to me how [intervention] will help [manage/improve] my [behaviour/condition/clinical outcome]” [42]).

#### Acceptability survey

The final survey was delivered online in English via Qualtrics, median completion time was 10.2 minutes. The survey comprised three sections as described below (see Supplementary material for full survey).

*i) Demographics.* This section collected demographic data including age, gender, employment status, income, and past or present mental illness diagnosis.

*ii) Information section.* As community awareness of lifestyle medicine and its applications vary widely, a brief information section was included that provided definitions of lifestyle medicine, mental illness, and examples of the use of lifestyle medicine to treat mental illness. The information also noted that lifestyle interventions could be undertaken with or without professional supports (i.e. independent behaviour change vs. with a dietitian, exercise physiologist, health coach). The information section was written by the research team and reviewed by an independent researcher for accuracy and unbiased phrasing.

*iii) Acceptability.* This section assessed the acceptability of lifestyle medicine, psychotherapy, and pharmacotherapy to treat mental illness. Each approach was rated across seven items, one for each component construct of the TFA. Participants indicated the extent to which they agreed or disagreed with each statement on a five-point Likert scale (1 = strongly disagree to 5 = strongly agree). As items assessing burden and opportunity cost were phrased negatively, responses to these items were reverse scored (i.e., strongly disagree became strongly agree, disagree became agree and vice versa) to ensure the directionality of responses across component constructs was comparable. Participants were then asked to rank five common lifestyle medicine activities (exercise, diet, sleep, social connection, and meditation) in order according to which they would most, to least likely engage with. Participants were also asked to rank the three treatment modalities according to which they would prefer to be prescribed if they were experiencing a mental illness.

### Data analysis

Data were analysed using Statistical Package for Social Sciences (SPSS) version 25.0. Descriptive statistics were used to document the acceptability of lifestyle medicine for treating mental illness across the seven TFA component constructs and to compare participants’ preferred lifestyle medicine activities and treatment modality (lifestyle medicine, pharmacotherapy, or psychotherapy). Mann-Whitney U tests were used to investigate differences in the acceptability of lifestyle medicine between people with and without a lived experience of mental illness. A series of Friedman’s repeated measures ANOVAs were used to examine differences in acceptability scores across the three treatment modalities for each TFA component construct. Wilcoxon signed-ranks test were used for post hoc analyses. An alpha of 0.05 was applied to all analyses.

## Results

### Participant characteristics

Of the 649 participants included in the analysis, 348 (53.6%) reported a past or present mental illness. Affective (47.2%) and anxiety disorders (41.9%) were the most prevalent mental illnesses and 231 participants reported experiencing more than one mental illness. Participants with and without lived experience of mental illness did not differ in age or gender, however those with a lived experience were more likely to be unemployed and have a lower income (Table 3). The majority of participants resided in east Australian states (Victoria (*n* = 320), New South Wales or Australian Capital Territory (*n* = 137) and Queensland (*n* = 80)), followed by Western Australia (*n* = 48), South Australia (*n* = 43), Northern Territory (*n* = 15), and Tasmania (*n* = 6).

**Table 3:** Participant Characteristics

Characteristic	Overall Sample (*n = 649)*	Lived Experience (*n* = 348)	No Lived Experience (*n* = 301)	
	*N (%) or mean (SD)*			*p*
Age	34.8 (12.7)	32.3 (11.1)	35.6 (13.4)	0.44
Gender				0.08
Male	234 (36.1)	110 (31.6)	124 (41.2)	
Female	406 (62.6)	232 (66.7)	174 (57.8)	
Non-binary/conforming	6 (0.9)	4 (1.1)	2 (0.7)	
Other	3 (0.5)	2 (0.6)	1 (0.3)	
Education				0.09
Primary school	2 (0.3)	2 (0.6)	64 (21.3)	
Secondary school	140 (21.6)	76 (21.8)	29 (9.6)	
TAFE	84 (12.9)	55 (15.8)	6 (2.0)	
Apprenticeship	17 (2.6)	11 (3.2)	133 (44.2)	
Bachelor’s	279 (43.0)	146 (42.0)	56 (18.6)	
Master’s	101 (15.6)	45 (12.9)	10 (3.3)	
Doctoral	22 (3.4)	12 (3.4)	3 (1.0)	
None of the above	4 (0.6)	1 (0.3)	64 (21.3)	
Employment				0.001
Employed	510 (78.6)	259 (74.4)	251 (83.4)	
Unemployed	82 (12.6)	58 (16.7)	24 (8.0)	
Retired	18 (2.8)	8 (2.3)	10 (3.3)	
Student	135 (20.8)	74 (21.3)	24 (8.0)	
Annual Income				0.001
$0 - $18,200	155 (23.9)	94 (27.0)	61 (20.3)	
$18,201 - $37,000	115 (17.7)	71 (20.4)	44 (14.6)	
$37,001 - $90,000	219 (33.7)	110 (31.6)	109 (36.2)	
$90,001 - $180,000	138 (21.3)	69 (19.8)	69 (22.9)	
$180,001 and over	22 (3.4)	4 (1.1)	18 (6.0)	
Experienced mental illness				
Yes	348 (53.6)			
No	301 (46.4)			
Mental Illness				
Affective disorders	307 (47.2)			
Anxiety disorders	272 (41.9)			
Obsessive Compulsive Disorder	31 (4.8)			
Substance Addiction	40 (6.2)			
Gambling Addiction	8 (1.2)			
Psychotic disorders	7 (1.1)			
Eating and Body Image Disorders	61 (9.5)			

*M* = mean, *SD* = standard deviation, *n* = frequency; % = percentage.

### Acceptability of lifestyle medicine

**Fig. 1:**
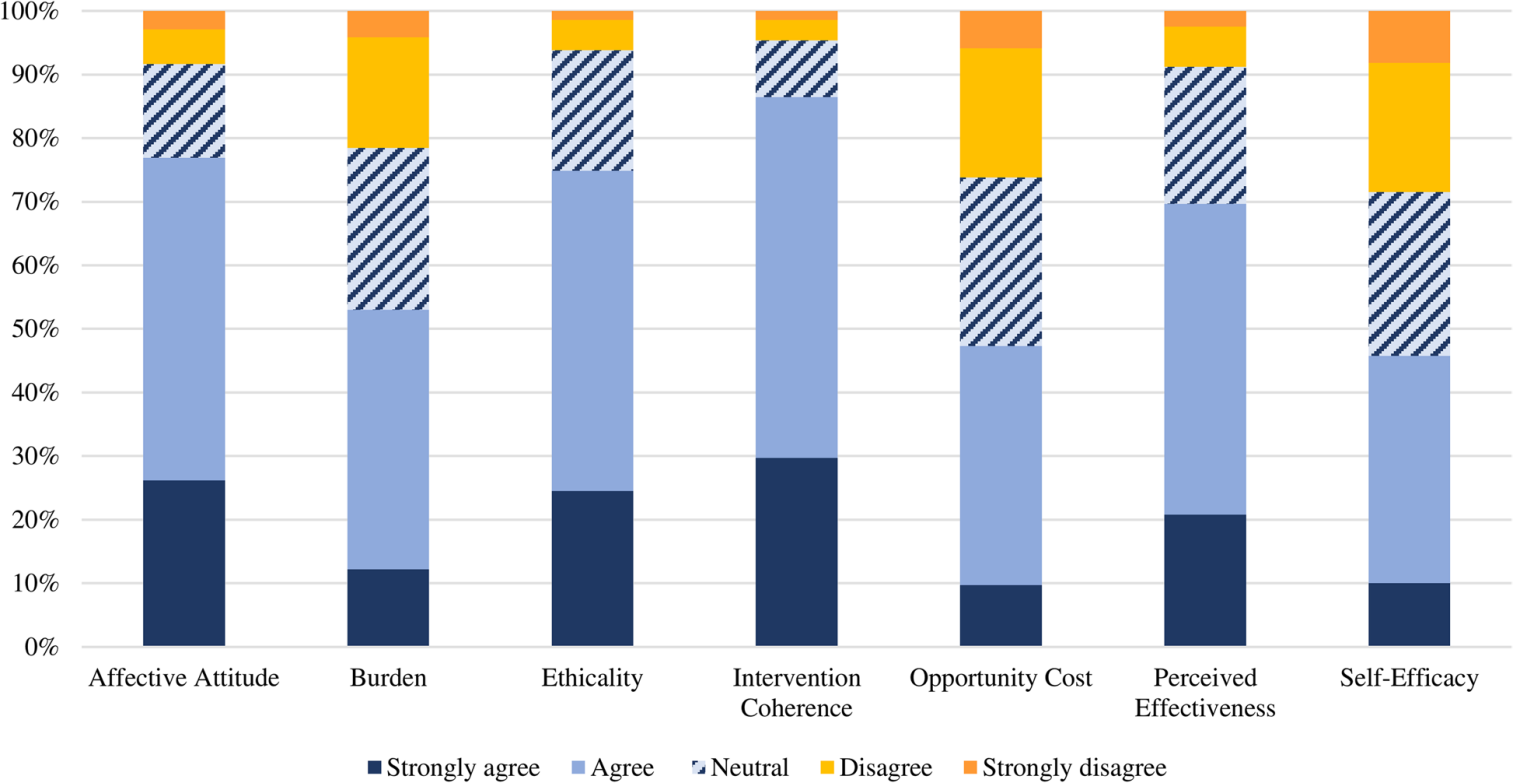
Acceptability of lifestyle medicine for treating mental illness. Items assessing burden and opportunity cost were reverse scored such that strongly agree and agree indicated that the burden and opportunity cost was acceptable to participants (i.e. lower scores indicate these constructs are less acceptable)

The majority of participants agreed or strongly agreed that they felt positive about lifestyle medicine (affective attitude = 76.9%), that this treatment approach aligned with their personal values (ethicality = 74.9%), they understood how lifestyle medicine would work (intervention coherence = 86.4%) and thought it would be effective (perceived effectiveness = 69.6%). The burden associated with engaging in lifestyle medicine was acceptable to fewer participants (53%). Less than half of participants reported that what they would have to give up in order to engage was acceptable (opportunity cost = 47.3%) or were confident in their ability to engage in lifestyle medicine activities (self-efficacy = 45.7%; Fig. 1). Across common lifestyle medicine activities with demonstrated efficacy for mental illness, participants indicated they would be most likely to engage in physical exercise (30.2%), followed by diet (14.5%), sleep (13.8%), social connection (9.3%), and meditation (3.9%). This pattern of preferences held for participants with lived experience of mental illness, however people without lived experience had a slight preference for sleep interventions (16.3%) over diet modification (15.9%).

### Effect of lived experience on the acceptability of lifestyle medicine

**Fig. 2:**
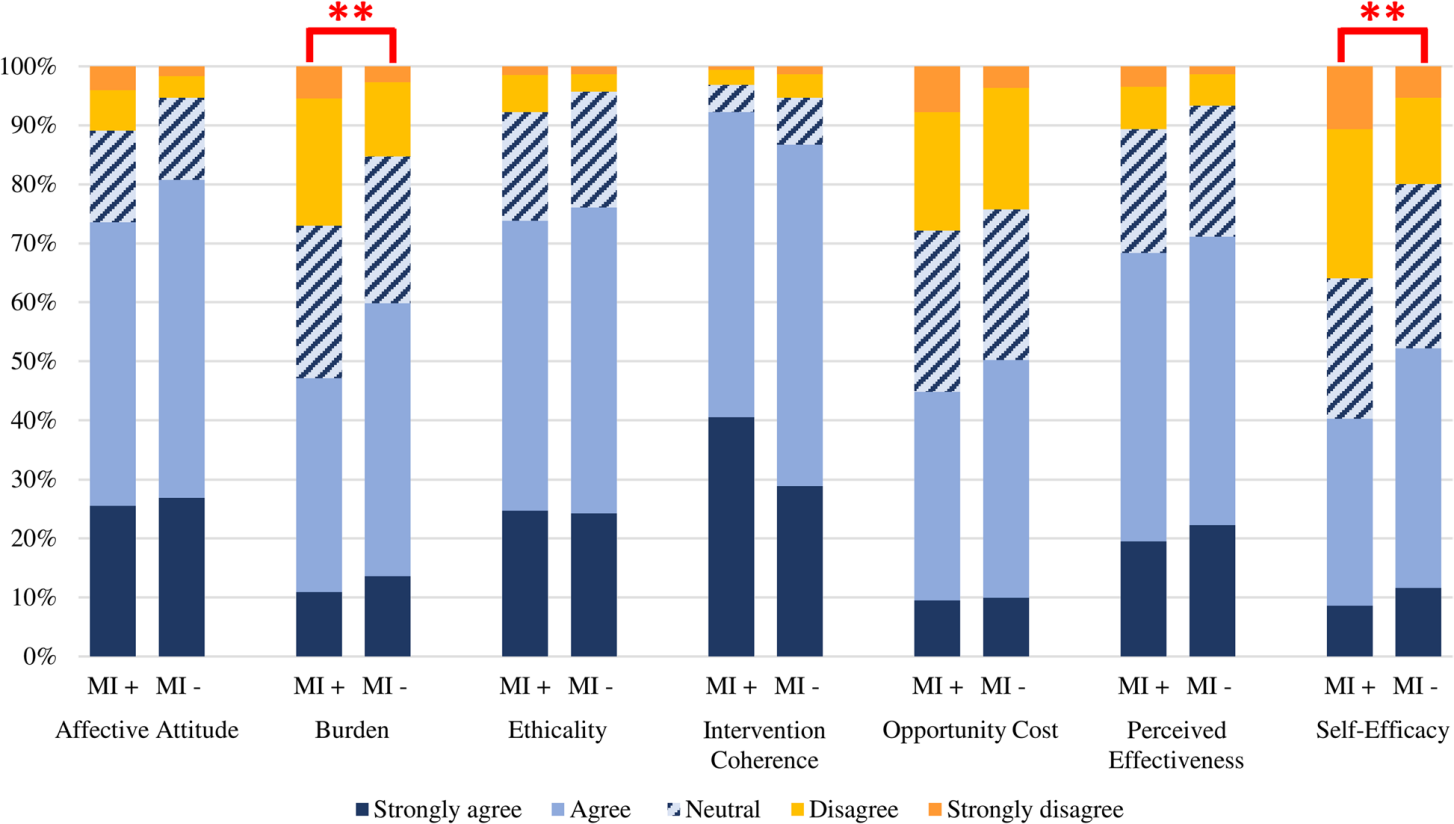
Acceptability of lifestyle medicine for people with and without lived experience of mental illness. Items assessing burden and opportunity cost were reverse scored such that strongly agree and agree indicated that the burden and opportunity cost was acceptable to participants (i.e. lower scores indicate these constructs are less acceptable). MI+ = participants with lived experience of mental illness; MI- = participants with no lived experience of mental illness. **p < 0.001

Responses to TFA items assessing burden and self-efficacy to engage in lifestyle medicine activities differed significantly between individuals with and without lived experience of a mental illness (Fig. 2, see supplementary materials Table S1 for full frequency statistics). The burden associated with engaging in lifestyle medicine was less acceptable to participants who had experienced mental illness than those who had not (*U =* 60,470*, z =* 3.57*, r =* 0.14*, p* < 0.001). Participants with lived experience also reported lower self-efficacy to engage in lifestyle medicine activities compared to participants without lived experience (*U =* 61,587*, z =* 4.02*, r =* 0.16*, p* < 0.001). No significant differences in responses to items assessing affective attitude, ethicality, intervention coherence, opportunity cost, or perceived effectiveness were observed (all *p* > 0.05).

### The acceptability of lifestyle medicine compared to psychotherapy and pharmacotherapy

**Fig. 3:**
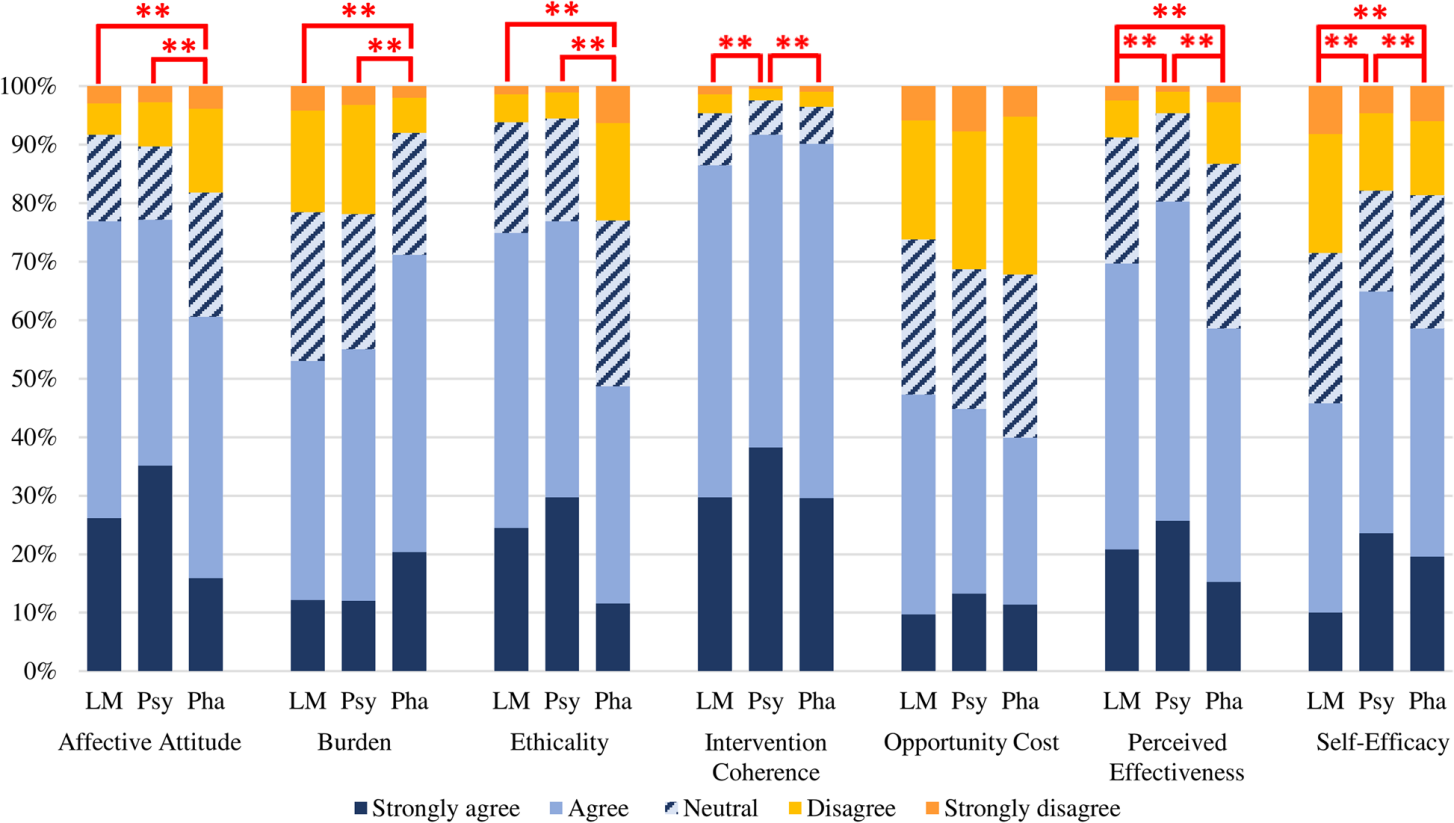
Acceptability of lifestyle medicine, psychotherapy and pharmacotherapy. Items assessing burden and opportunity cost were reverse scored such that strongly agree and agree indicated that the burden and opportunity cost was acceptable to participants. (i.e. lower scores indicate these constructs are less acceptable). LM = Lifestyle medicine; Psy = Psychotherapy, Pha = Pharmacotherapy. ** *p* < .001

Lifestyle medicine (46.8%) was most frequently ranked as participant’s preferred treatment modality, followed by psychotherapy (37.6%) and pharmacotherapy (15.6%). This order of preferences held for participants without lived experience of mental illness, however, people with lived experience preferred psychotherapy (41.7%) followed by lifestyle medicine (34.8%). The distribution of participant responses was significantly different between the three treatment modalities for the TFA component constructs of affective attitude (*X*^*2*^ (2) *86.20, = p* < 0.001), burden (*X*^*2*^ (2) *86.71, = p* < 0.001), ethicality (*X*^*2*^ (2) *= 215.60, = p* < 0.001), intervention coherence (*X*^*2*^ (2) *30.45, = p* < 0.001), perceived effectiveness (*X*^*2*^ (2) *87.39, = p* < 0.001), and self-efficacy (*X*^*2*^ (2) *80.18, = p* < 0.001). There were no significant differences in opportunity cost (Fig. 3, see supplementary materials Table S2 for full frequency statistics). Participants felt more positive (affective attitude) towards lifestyle medicine (*M* = 3.92, *Z* = − 6.48, *p* < 0.001) and psychotherapy (*M* = 3.99, *Z* = − 8.44, *p* < 0.001) than pharmacotherapy (*M* = 3.54) and reported these approaches to be more in line with their personal values (ethicality; *Z* = − 10.24, *p* < 0.001 and *Z* = − 12.50, *p* < 0.001, respectively). In contrast pharmacotherapy (*M* = 3.54) was perceived as significantly less burdensome than lifestyle medicine (*M* = 3.54, *Z* = − 8.27, *p* < 0.001) and psychotherapy (*M* = 3.54, *Z* = − 8.44, *p* < 0.001). Intervention coherence ratings were significantly higher for psychotherapy (*M* = 4.27) than lifestyle medicine (*M* = 4.10, *Z* = − 4.97, *p* < 0.001) and pharmacotherapy (*M* = 4.15, *Z* = − 3.84, *p* < 0.001). Pharmacotherapy (*M* = 3.58) was perceived as less effective than lifestyle medicine (*M =* 4.00, *Z* = − 4.03, *p* < 0.001) and psychotherapy (*M =* 4.00, *Z* = − 9.71, *p* < 0.001), while lifestyle medicine was perceived as less effective than psychotherapy (*Z* = − 5.18, *p* < 0.001). Finally, self-efficacy differed significantly between all treatment modalities (all *p* < 0.001) such that participants were most confident in their ability to engage in psychotherapy (*M* = 3.66), followed by pharmacotherapy (*M* = 3.53) and lifestyle medicine (*M* = 3.19).

## Discussion

The current study is the first to investigate the acceptability of lifestyle medicine for treating mental illness and explore differences in perspectives across people with and without lived experience of mental illness. The broad pattern of acceptability was such that participants typically found the more emotive (affective attitude, ethicality) and cognitive (intervention coherence, perceived effectiveness) component constructs of the TFA to be highly acceptable. In contrast, across component constructs that reflect the practicalities of engaging in lifestyle medicine (burden, opportunity cost, and self-efficacy), acceptability was comparatively lower. Participants with lived experience of mental illness viewed lifestyle medicine as more burdensome and were less confident in their ability to engage, than those who had not experienced mental illness. Across the whole sample, participants felt more positive about (affective attitude) lifestyle medicine and psychotherapy, and that these two approaches were more in line with their values (ethicality) than pharmacotherapy. Pharmacotherapy, however, was perceived as less burdensome than the non-pharmacological treatments, and participants were least confident in their ability to engage in lifestyle medicine.

That participants generally felt positive about lifestyle medicine, believed it aligned with their values, understood how lifestyle medicine could treat mental illness, and perceived it to be an effective treatment option, indicate a favourable attitude towards this approach within the community. However, approximately half of participants felt that adopting lifestyle medicine activities would demand considerable effort, necessitate sacrificing other important priorities, and present challenges due to low self-efficacy, all of which would likely impede engagement to some degree. This highlights the need to support people to overcome barriers to initiating and maintaining health behaviour change. Behavioural science evidence indicates that group-based programs, supervised by an allied health professional (e.g. exercise physiologist or dietitian), alongside individualised motivational support, are most successful in establishing long-term health behaviour change [16, 23, 43] and therefore promoting mental health outcomes. However, referrals to allied health professionals for mental illness are rare, despite lifestyle change being recommended by peak bodies in psychiatry as non-negotiable first-line treatment for numerous mental health indications [7]. In Australia for example, 61% of primary health care visits for a mental health indication result in a prescription for medication versus less than 3% for lifestyle modification [44]. A lack of education and training is a known contributor to low referral rates by primary health care providers [45]. Furthermore, when referrals do occur, there is significant variability in allied health professionals’ knowledge of the best practice approaches for mental health indications and evidence-based programs specifically designed to assist people with mental illness in overcoming barriers to behaviour change are scarce [10].

The acceptability of lifestyle medicine did not differ between people with and without personal experience of mental illness across affective attitude, ethicality, intervention coherence, or perceived effectiveness. People with lived experience, did however, report lower self-efficacy to engage in lifestyle medicine. This is of particular importance as self-efficacy is a known predictor for the adoption and maintenance of healthy lifestyle behaviours [46–48]. To be maximally successful interventions should therefore embed strategies to increase self-efficacy. For example, providing opportunities for people to experience mastery has been shown to improve self-efficacy and adherence to lifestyle interventions [49–51]. Participants with lived experienced also felt that engaging in lifestyle medicine would be more burdensome. This may reflect the difficulties people face when attempting lifestyle changes while experiencing mental health symptoms (e.g. low energy, anhedonia, reduced motivation). It suggests that people who have experienced mental illness have a deeper understanding of the challenges associated with making health behaviour change while unwell, and are likely to require greater practical and psychological support to feel empowered and capable of engaging in sustained lifestyle change. Given a long history of researchers developing treatments without consulting end-users, and the potential downstream consequences for adherence, these findings highlight the importance of engaging people with experience of mental illness in the co-design of lifestyle-based interventions to ensure that such programs meet their wants and needs.

When comparing treatment modalities, the was a clear preference for psychotherapy and lifestyle medicine over medication. These findings are consistent with an existing body of research showing widespread preference for non-medication treatments in psychiatry and indicate that this perspective has not changed in over 20 years [52, 53]. While previous studies have primarily compared psychotherapy to pharmacotherapy [53], the results of the current study extend this work by also demonstrating a preference for lifestyle medicine. It highlights an ongoing clinical discrepancy that, for many, the least desired treatment approach is the most widely available and commonly prescribed [44]. While there has been global acknowledgement of the need to increase patient choice [54], this shift has yet to occur at scale despite evidence that receiving a preferred treatment may result in greater treatment compliance, additional therapeutic benefit, and increase cost-effectiveness [53, 54].

The use of the TFA enabled insight into which facets of acceptability may be driving treatment preferences. Participants felt more positive about and ethically aligned with the use of lifestyle medicine and psychotherapy than pharmacotherapy. They also perceived psychotherapy, followed by lifestyle medicine, as being more effective than pharmacotherapy in treating mental illness. That lifestyle medicine was widely perceived to be effective is notable, given how recent and evolving the use of behavioural interventions for mental health is. While perceived understanding of *how* each treatment might work (intervention coherence) was highest (91.7%) for psychotherapy, the degree to which this was better understood than lifestyle medicine (86.4%) or pharmacology (90.2%) was modest. In comparison, pharmacotherapy was viewed as being less burdensome than both lifestyle medicine and psychotherapy. This is unsurprising given the substantial time and effort frequently associated with engaging in these activities [55] compared with medication. Finally, participants felt they would need to give up something (opportunity cost) in order to engage in any treatment for mental illness. Time and cost are commonly cited as barriers to engaging in lifestyle medicine and psychotherapy [55–57], and medication use may be associated with side-effects [58], possibly resulting in a need to sacrifice other aspects of health. However, additional qualitative research is needed to provide a greater understanding of the factors underlying this finding.

The current findings have implications for the design and implementation of lifestyle medicine interventions into mental healthcare. While a preference for lifestyle medicine for mental illness was observed, and many components of acceptability were high, there are also clear barriers to engaging in lifestyle medicine. Going forward, harnessing gold-standard frameworks for behavioural intervention design and development (e.g., The Behaviour Change Wheel [59], ORBIT [60]) is likely to be particularly valuable in identifying and systematically addressing barriers to change. The sustainability and scalability of lifestyle medicine programs should also be considered. Leveraging the rise in digital health innovations may offer an avenue for greater scalability; however, given the efficacy professional support offers, combined approaches may balance the need for sustainability, scalability and personalisation, and ultimately lead to greater improvements in mental health outcomes. Although understanding acceptability to end-users is essential, documenting how acceptable lifestyle-based therapeutics are to clinicians (general practitioners, psychologists, psychiatrists, allied health clinicians), service managers, and payers (government and private) is also necessary. As gatekeepers to mental healthcare services and policy, the combined perspectives of these key stakeholders will be essential to facilitate system-level change.

The current data should be considered in context of a number of strengths and limitations. The use of the TFA provided a rich multifaceted breakdown of acceptability, enabling a nuanced appreciation of community attitudes to lifestyle medicine for mental illness and how this compares with current first-line treatments. It was, however, restricted to the assessment of *prospective *(before treatment) acceptability in the context of the examples provided, without considering participants’ experience with the various treatment approaches. First-hand experience of these treatments, whether as a patient or clinician, will inevitably alter perspectives. This study also explored the acceptability of lifestyle medicine as a broad category of approaches. Given the observed preference for specific lifestyle activities (i.e., exercise, diet, and sleep), it will be valuable to assess *concurrent* (during treatment) and *retrospective* (after treatment) acceptability of specific lifestyle interventions. Lastly, the current study did not account for potential variations in acceptability based on type of mental health diagnosis due to the diverse nature of the sample. Further research would be necessary to explore potential interactions between diagnosis and the acceptability of different lifestyle medicine approaches.

## Conclusion

There is a compelling body of evidence demonstrating the effectiveness of lifestyle medicine approaches in treating mental illness. Given the current growth in the field, now is the time to progress the development of integrated behavioural approaches and systematic implementation into mental health care. Understanding the acceptability of interventions is a crucial initial step towards achieving this. The current findings illustrate a preference for lifestyle medicine for treating mental illness and indicate a need for healthcare infrastructure to support programs that help people initiate and, most importantly, maintain healthy lifestyle change. Designing and implementing interventions specifically for people with mental illness that address the burden and opportunity cost of engaging in lifestyle medicine and increase self-efficacy will be particularly powerful.

### Supplementary Information

**Additional file 1.**

## Data Availability

The datasets used and/or analysed during the current study are available from the corresponding author on reasonable request.
