# Supplementary material for: The acceptability of lifestyle medicine for the treatment of mental illness: perspectives of people with and without lived experience of mental illness
Source: BMC Public Health. 2024 Jan 13;24:171. doi: 10.1186/s12889-024-17683-y (PMC10787508; doi:10.1186/s12889-024-17683-y)
Supplement: Supplementary file 1 — Additional file 1. [file 12889_2024_17683_MOESM1_ESM.docx]

**Supplementary Materials**

Acceptability Survey

**Demographic Questions**

1a) How would you describe your gender?

- Male
- Female
- Non-binary/Non-conforming
- Other

1b) What is your age?

1c) How would you describe your ethnicity?

1d) What is the highest level of education you have completed?

- Primary/elementary school
- Secondary school
- TAFE
- Apprenticeship
- Bachelor or equivalent
- Masters or equivalent
- Doctoral or equivalent
- None of the above

1e) Please enter your postcode:

1f) How would you describe your current employment status? (Please select all that apply)

- Part-time
- Full-time
- Self-employed
- Contractor
- Permanent
- Casual
- Student
- Unemployed
- Retired

1g) What is your annual personal income?

- $0 - $18,200
- $18,201 - $37,000
- $37,001 - $90,000
- $90,001 - $180,000
- $180,001 and over

1h) Have you ever been diagnosed with a mental illness by your GP, a psychologist, psychiatrist or other medical professional?

- Yes
- No

Which conditions have been relevant for you? (Please select all the apply)

|  | Past | Present |
| --- | --- | --- |
| Depression |  |  |
| Bipolar disorder |  |  |
| Anxiety disorder |  |  |
| Obsessive compulsive disorder |  |  |
| Schizophrenia or other psychotic condition |  |  |
| Post-traumatic stress disorder |  |  |
| Alcohol addiction |  |  |
| Drug addiction |  |  |
| Gambling addiction |  |  |
| Anorexia |  |  |
| Bulimia |  |  |
| Other (please describe below) |  |  |

**2. INFORMATION SECTION**

*Please read the following descriptions of mental illness and lifestyle medicine, the questions below relate to these descriptions.*


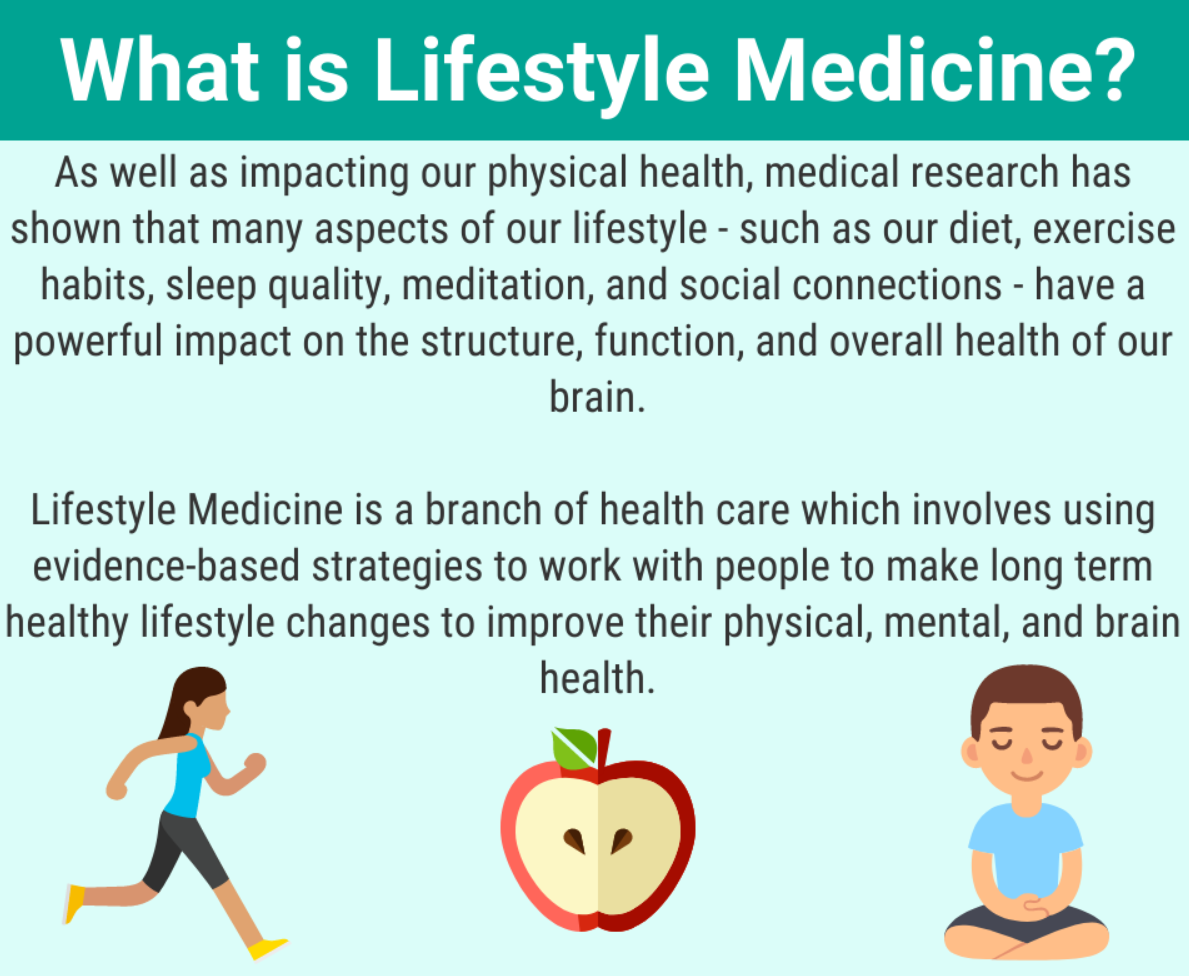


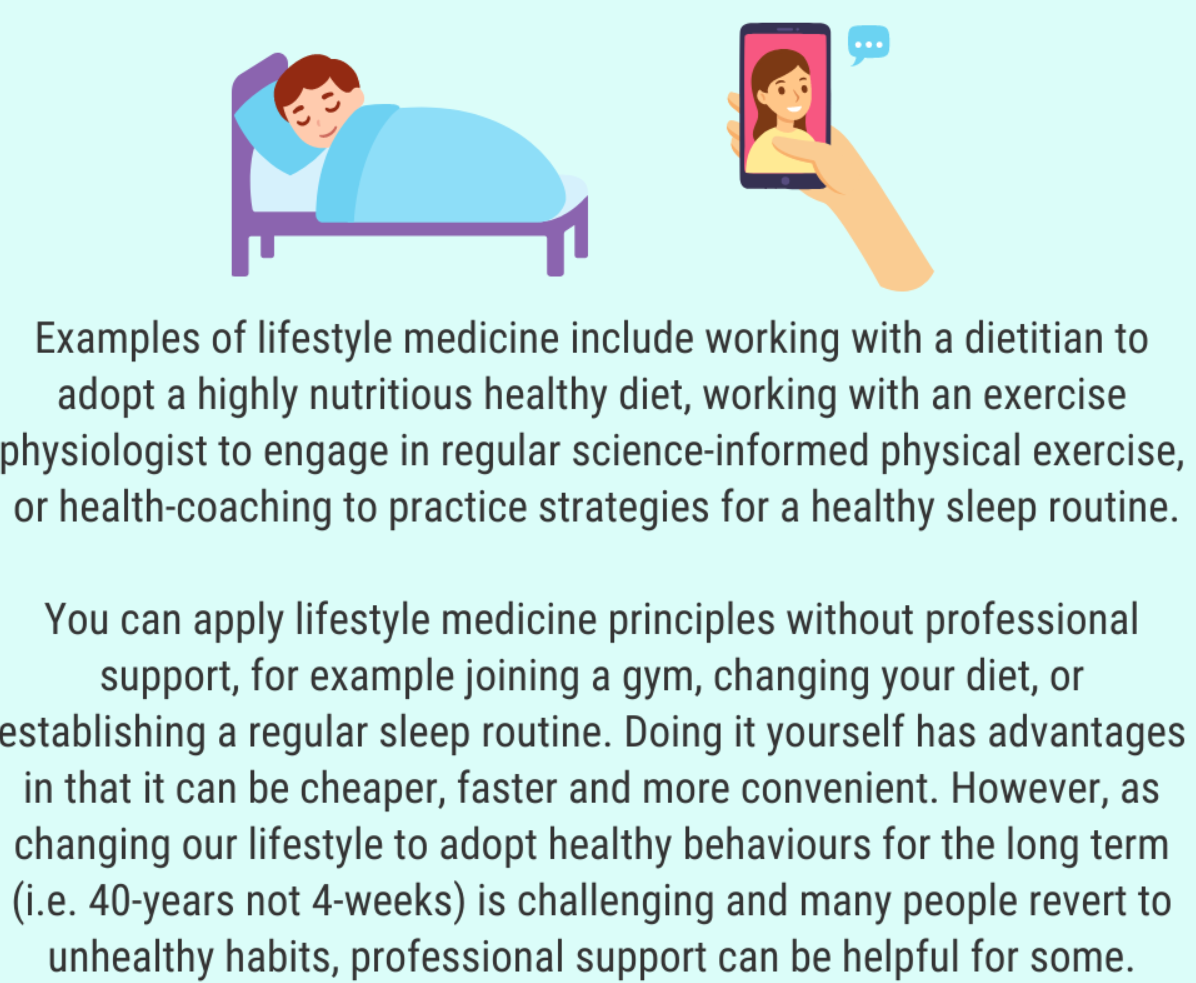


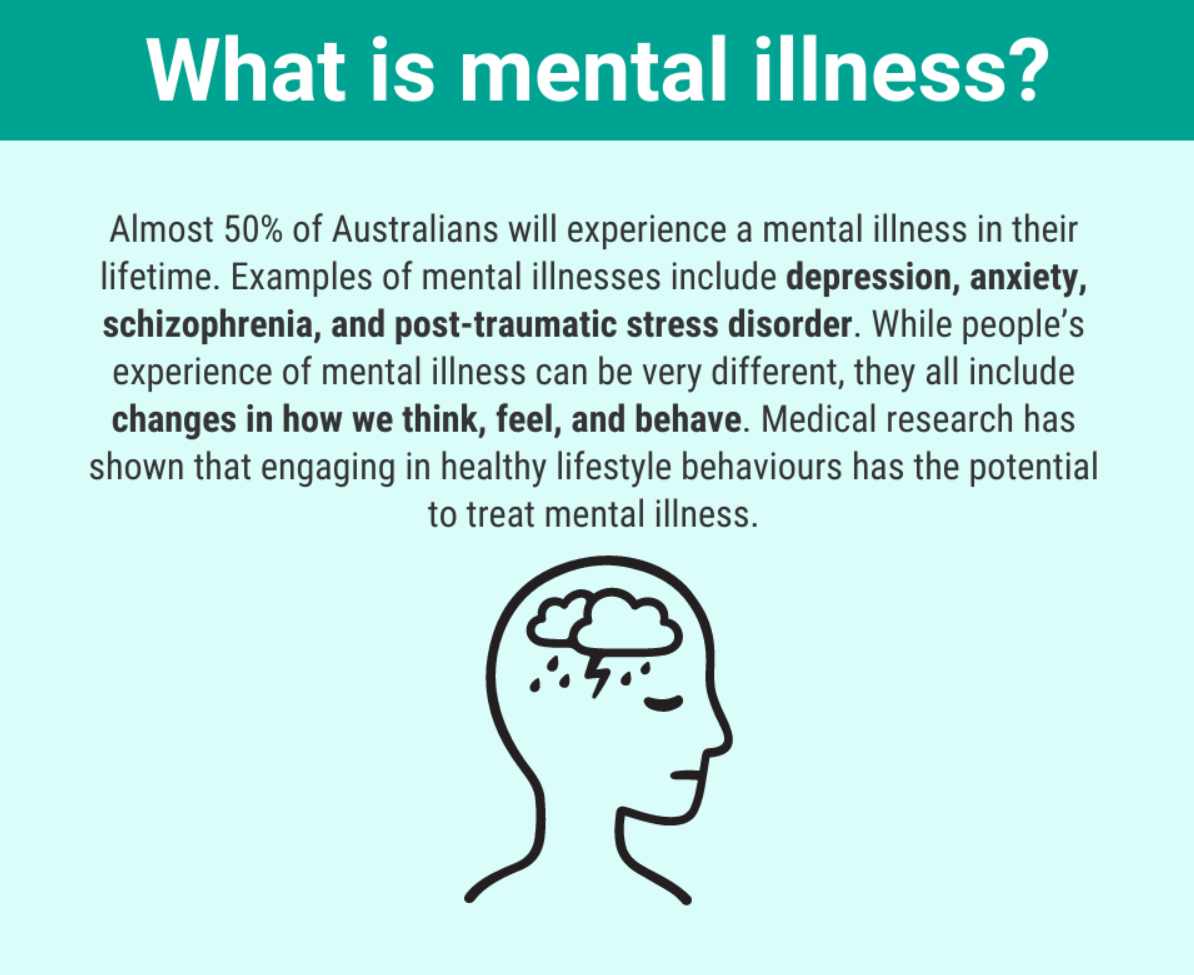


**3. ACCEPTABILITY SECTION**

Thinking about the use of lifestyle medicine activities for **enhancing mental health**, please indicate to what extent you agree or disagree with the following statements.

- I feel positive about the use of lifestyle medicine to treat mental illness
- I think engaging in lifestyle medicine to treat mental illness would require too much effort
- The use of lifestyle medicine to treat mental illness fits with my personal values
- I understand how engaging in lifestyle medicine activities could treat mental illness
- Engaging in lifestyle medicine activities to treat mental illness would come at a cost, it would mean giving up other things that are important to me
- Engaging in regular lifestyle medicine activities would be an effective treatment for mental illness
- If I had a mental illness, I am confident I could regularly engage in lifestyle medicine activities to treat it

There are lots of different lifestyle medicine activities people can do to **treat mental illness**.

Please rank the following activities from the one you would be most likely to do (1 = most likely) then in descending order to the one you would be least likely to do (5 = least likely).

______ Regular physical

______ Maintaining a healthy diet

______ Working on your connection with others

______ Maintaining a healthy sleep pattern

______ Practice regular meditation

Thinking about the use of medication to **treat mental illness**, please indicate to what extent you agree or disagree with the following statements.

- I feel positive about the use of medication to treat mental illness
- I think taking medication to treat mental illness would require too much effort
- The use of medication to treat mental illness fits with my personal values
- I understand how taking medication could treat mental illness
- Taking medication to treat mental illness would come at a cost, it would mean giving up other things that are important to me
- Taking medication regularly would be an effective treatment for mental illness

Thinking about seeing a psychologist to **treat mental illness**, please indicate to what extent you agree or disagree with the following statements.

- I feel positive about seeing a psychologist to treat mental illness
- I think seeing a psychologist to treat mental illness would require too much effort
- Seeing a psychologist to treat mental illness fits with my personal values
- I understand how seeing a psychologist could treat mental illness
- Seeing a psychologist to treat mental illness would come at a cost, it would mean giving up other things that are important to me
- Seeing a psychologist regularly would be an effective treatment for mental illness
- If I had a mental illness, I am confident I could see a psychologist regularly to treat it

If you were experiencing a **mental illness** which of the following **treatment approaches** would you prefer?

*Please rank the following treatments from the one you would most prefer (1 = most prefer) then in descending order to the one you would least prefer (3 = least prefer).*

- Lifestyle medicine
- Seeing a psychologist
- Medication

Thank you for completing the survey!

Your contribution will help us better understand the acceptability of lifestyle medicine within the community.

If answering these survey questions raised any concerns for you regarding your brain health we recommend speaking to your GP or health professional and see below for some additional support resources:

Lifeline Australia – immediate mental health and crisis support, and suicide prevention 
[https://www.lifeline.org.au/](http://https/www.lifeline.org.au/)
13 11 14

Turning Point - alcohol, drug, gambling (and other behavioural concerns) support 
<https://www.turningpoint.org.au/>

We would really appreciate it if you could share this survey with your friends and family so that we can include a broad range of people in our study.
If you wish to share this survey, please copy and paste the following link:

[Healthy Lifestyles for Healthy Minds](https://monash.az1.qualtrics.com/jfe/form/SV_8eau8lFjZLs0UCN)

Or you can share on Facebook, Instagram, or Twitter by clicking on the icons below...
[
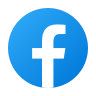
](https://www.facebook.com/BrainParkMonash/posts/690573861520539) [
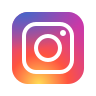
](https://www.instagram.com/p/CCaG-vQDJwK/) [
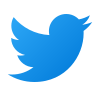
](https://twitter.com/BrainPark/status/1281082271726891008)

If you want to enter in the draw to win one of three $50 Coles/Myer gift vouchers please follow the link below.

[Prize Draw Entry](https://monash.az1.qualtrics.com/jfe/form/SV_3ELsBGTIWLBPkOx)

This is **voluntary**and not required as part of the survey, you can close the window if you do not wish to enter the prize draw

**Table S1.** A*cceptability of lifestyle medicine for people with and without lived experience of mental illness*

| TFA Component  Construct | | Strongly Disagree | Disagree | Neither agree nor disagree | Agree | Strongly Agree |
| --- | --- | --- | --- | --- | --- | --- |
|  | | % | % | % | % | % |
| Affective Attitude | MI+ | 4.0 | 6.9 | 15.5 | 48 | 25.6 |
|  | MI- | 1.7 | 3.7 | 14.0 | 53.8 | 26.9 |
|  |  |  |  |  |  |  |
| Burden | MI+ | 5.5 | 21.6 | 25.9 | 36.2 | 10.9 |
|  | MI- | 2.7 | 12.6 | 24.9 | 46.2 | 13.6 |
|  |  |  |  |  |  |  |
| Ethicality | MI+ | 1.4 | 6.3 | 18.4 | 49.1 | 24.7 |
|  | MI- | 1.3 | 3.0 | 19.6 | 51.8 | 24.3 |
|  |  |  |  |  |  |  |
| Intervention coherence | MI+ | 1.4 | 2.6 | 9.8 | 55.7 | 30.5 |
|  | MI- | 1.3 | 4.0 | 8.0 | 57.8 | 28.9 |
|  |  |  |  |  |  |  |
| Opportunity Cost | MI+ | 7.8 | 20.1 | 27.3 | 35.3 | 9.5 |
|  | MI- | 3.7 | 20.6 | 25.6 | 40.2 | 10.0 |
|  |  |  |  |  |  |  |
| Perceived Effectiveness | MI+ | 3.4 | 7.2 | 21.0 | 48.9 | 19.5 |
|  | MI- | 1.3 | 5.3 | 22.3 | 48.8 | 22.3 |
|  |  |  |  |  |  |  |
| Self-Efficacy | MI+ | 10.6 | 25.3 | 23.9 | 31.6 | 8.6 |
|  | MI- | 5.3 | 14.6 | 27.9 | 40.5 | 11.6 |

*Note.* MI+ = lived experience of mental illness; MI- = no lived experience of mental illness

**Table S2.** *Participant responses (%) to acceptability items by treatment modality*

| TFA component construct | | Strongly Disagree | Disagree | Neither agree nor disagree | Agree | Strongly Agree |
| --- | --- | --- | --- | --- | --- | --- |
|  | | % | % | % | % | % |
| Affective  Attitude | LM | 2.9 | 5.4 | 14.8 | 50.7 | 26.2 |
|  | Pha | 3.9 | 14.3 | 21.3 | 44.7 | 15.9 |
|  | Psy | 2.8 | 7.6 | 12.5 | 42.1 | 35.1 |
|  |  |  |  |  |  |  |
| Burden | LM | 4.2 | 17.4 | 25.4 | 40.8 | 12.2 |
|  | Pha | 2.0 | 6.0 | 20.8 | 50.8 | 20.3 |
|  | Psy | 3.2 | 18.6 | 23.1 | 43.0 | 12.0 |
|  |  |  |  |  |  |  |
| Ethicality | LM | 1.4 | 4.8 | 19 | 50.4 | 24.5 |
|  | Pha | 6.3 | 16.6 | 28.4 | 37.1 | 11.6 |
|  | Psy | 1.1 | 4.5 | 17.6 | 47.1 | 29.7 |
|  |  |  |  |  |  |  |
| Intervention coherence | LM | 1.4 | 3.2 | 8.9 | 56.7 | 29.7 |
|  | Pha | 0.9 | 2.6 | 6.3 | 60.6 | 29.6 |
|  | Psy | 0.5 | 2.0 | 5.0 | 53.5 | 38.2 |
|  |  |  |  |  |  |  |
| Opportunity  Cost | LM | 5.9 | 20.3 | 26.5 | 37.6 | 9.7 |
|  | Pha | 5.2 | 27.0 | 27.9 | 28.5 | 11.4 |
|  | Psy | 7.7 | 23.6 | 23.9 | 31.6 | 13.3 |
|  |  |  |  |  |  |  |
| Perceived Effectiveness | LM | 2.5 | 6.3 | 21.6 | 48.8 | 20.8 |
|  | Pha | 2.8 | 10.5 | 28.2 | 43.3 | 15.3 |
|  | Psy | 0.9 | 3.7 | 15.1 | 54.5 | 25.7 |
|  |  |  |  |  |  |  |
| Self-Efficacy | LM | 8.2 | 20.3 | 25.7 | 35.7 | 10 |
|  | Pha | 6.0 | 12.6 | 22.8 | 39.0 | 19.6 |
|  | Psy | 4.6 | 13.3 | 17.3 | 41.3 | 23.6 |

*Note.* LM = lifestyle medicine; Pha = pharmacotherapy; Psy = psychotherapy
